# Clinical predictors of response to methotrexate in patients with rheumatoid arthritis: a machine learning approach using clinical trial data

**DOI:** 10.1186/s13075-022-02851-5

**Published:** 2022-07-01

**Authors:** Stephanie Q. Duong, Cynthia S. Crowson, Arjun Athreya, Elizabeth J. Atkinson, John M. Davis, Kenneth J. Warrington, Eric L. Matteson, Richard Weinshilboum, Liewei Wang, Elena Myasoedova

**Affiliations:** 1 Department of Quantitative Health Sciences, Mayo Clinic, Rochester, MN USA; 2 Division of Rheumatology, Department of Internal Medicine, Mayo Clinic, Rochester, MN USA; 3 Department of Molecular Pharmacology and Experimental Therapeutics, Mayo Clinic, Rochester, MN USA

**Keywords:** Rheumatoid arthritis, Methotrexate, Treatment, Machine learning

## Abstract

**Background:**

Methotrexate is the preferred initial disease-modifying antirheumatic drug (DMARD) for rheumatoid arthritis (RA). However, clinically useful tools for individualized prediction of response to methotrexate treatment in patients with RA are lacking. We aimed to identify clinical predictors of response to methotrexate in patients with rheumatoid arthritis (RA) using machine learning methods.

**Methods:**

Randomized clinical trials (RCT) of patients with RA who were DMARD-naïve and randomized to placebo plus methotrexate were identified and accessed through the Clinical Study Data Request Consortium and Vivli Center for Global Clinical Research Data. Studies with available Disease Activity Score with 28-joint count and erythrocyte sedimentation rate (DAS28-ESR) at baseline and 12 and 24 weeks were included. Latent class modeling of methotrexate response was performed. The least absolute shrinkage and selection operator (LASSO) and random forests methods were used to identify predictors of response.

**Results:**

A total of 775 patients from 4 RCTs were included (mean age 50 years, 80% female). Two distinct classes of patients were identified based on DAS28-ESR change over 24 weeks: “good responders” and “poor responders.” Baseline DAS28-ESR, anti-citrullinated protein antibody (ACPA), and Health Assessment Questionnaire (HAQ) score were the top predictors of good response using LASSO (area under the curve [AUC] 0.79) and random forests (AUC 0.68) in the external validation set. DAS28-ESR ≤ 7.4, ACPA positive, and HAQ ≤ 2 provided the highest likelihood of response. Among patients with 12-week DAS28-ESR > 3.2, ≥ 1 point improvement in DAS28-ESR baseline-to-12-week was predictive of achieving DAS28-ESR ≤ 3.2 at 24 weeks.

**Conclusions:**

We have developed and externally validated a prediction model for response to methotrexate within 24 weeks in DMARD-naïve patients with RA, providing variably weighted clinical features and defined cutoffs for clinical decision-making.

**Supplementary Information:**

The online version contains supplementary material available at 10.1186/s13075-022-02851-5.

## Background

Methotrexate (MTX) is the preferred initial disease-modifying antirheumatic drug (DMARD) for rheumatoid arthritis (RA). While MTX is the only drug needed to control RA disease activity for many patients with RA, up to 50% of patients respond inadequately to MTX and require additional treatments [1]. A 3- to 6-month trial of MTX treatment is generally recommended before a decision is made regarding MTX efficacy [2]. This delay can result in missing the window of opportunity for effective treatment of RA disease activity and unnecessary exposure to potential MTX-related side effects. Response to therapy in the first 6 months of RA diagnosis correlates with long-term outcomes [3], offering a strong rationale for identifying early predictors of treatment response.

Longer RA disease duration, higher baseline disease activity score including 28 joints (DAS28), female sex, younger age, smoking, and alcohol consumption have been associated with a lower likelihood of treatment success with MTX in observational studies and clinical trials [4–10]. These predictors vary depending on the definition of treatment response, i.e., achieving a state of remission or low disease activity versus absolute improvement in disease activity metrics, and there is a lack of a uniform and clinically useful prediction model of treatment response to MTX [11].

The use of machine learning (ML) in data analysis to inform individualized clinical decision-making and improve patient outcomes is on the rise across the spectrum of medical specialties, including rheumatology [12–14]. A recent large observational study of MTX-naïve patients with early RA (*n* > 5000) showed that ML methods integrating baseline clinical data did not significantly improve the prediction of MTX treatment persistence at 12 months compared to manual modeling, and the highest area under the curve (AUC) for least absolute shrinkage and selection operator (LASSO) regression was only 0.67 [15]. Smaller observational studies (*n* = 355) showed that the performance of ML algorithms (LASSO models AUC 0.76) was not superior to the logistic regression (AUC 0.77) in predicting DAS28 > 3.2 at 3 months of treatment in patients with RA who used MTX as monotherapy or in combination with other DMARDs [16]. Whether ML can provide a robust and clinically useful prediction of response to MTX monotherapy in the first months of treatment in patients with early RA using uniformly collected baseline demographics and clinical data has not been investigated in large patient populations.

To address this knowledge gap in RA management, we applied ML methods to randomized clinical trial (RCT) data to (1) algorithmically identify the classes of patients with RA and distinct trajectories of their DAS28 erythrocyte sedimentation rate (DAS28-ESR) from baseline to week 24, (2) identify the clinical predictors of belonging to one versus the other class(es) with external validation of the model, and (3) identify the predictors of achieving DAS28-ESR ≤ 3.2 at 24 weeks among patients with incomplete response, i.e., DAS28-ESR > 3.2 at week 12.

We hypothesized that patients with RA have distinct trajectories of response to MTX based on DAS28-ESR in the first 24 weeks of treatment and that response to MTX can be reliably predicted using a combination of baseline clinical data with the highest predictive importance, with validation in an independent dataset.

## Methods

### Patients

Four RCTs enrolling MTX-naïve patients who were randomized to placebo plus MTX were identified retrospectively through the Clinical Study Data Request Consortium (CSDR) via the Vivli Center for Global Clinical Research Data (Additional file 1: Table S1).

### Outcome and characteristics

Response to MTX was measured by the DAS28-ESR at baseline and 12 and 24 weeks. Pretreatment baseline characteristics included sociodemographics (age, sex, race), baseline DAS28-ESR and its individual parameters (i.e., 28 tender joint count (TJC28), 28 swollen joint count (SJC28), erythrocyte sedimentation rate (ESR), and patient global assessment of disease activity (PtGA)), C-reactive protein (CRP), physician global assessment of disease activity (PhGA), RA duration, baseline use of glucocorticoids, Health Assessment Questionnaire (HAQ) score, and serologic status: positive for rheumatoid factor (RF) or anti-citrullinated protein antibodies (ACPA).

### Statistical analysis

Latent class mixed modeling was applied to define patient classes with distinct DAS28-ESR trajectories using all available data. Two randomly selected RCTs were used in model development (i.e., training), and the remaining two RCTs were used for validation of the built model (i.e., testing). Baseline characteristics between good and poor responder groups defined using latent class mixed modeling as well as training and test sets were compared using the Kruskal–Wallis rank sum tests and Pearson’s chi-squared tests. Two models were assessed. The first is the DAS28-ESR model (i.e., all baseline characteristics except individual components of the DAS28-ESR, CRP, and PhGA), and the second consisted of components of DAS28-ESR (i.e., all characteristics except baseline DAS28-ESR). Missing values in predictor data were imputed using proximity from the randomForest package [17], and missing data indicator variables were created and included in the statistical models of interest. LASSO and random forests supervised classification methods were implemented to identify predictors of good response to MTX treatment. LASSO is a penalized regression method that shrinks the size of the coefficients to reduce the effective degrees of freedom in the model [18, 19]. The random forests methods rely on the framework of a tree-based model—a set of if–then rules to generate predictions from one or more decision trees—and averages predictions over an ensemble of many individual trees created with bootstrap samples of the dataset [20]. A random seed was set for reproducibility and tenfold cross-validation was performed to arrive at the final LASSO model using the minimum lambda while utilizing default settings and configurations for the implementation of random forests with 500 trees and settings based on the square root of the number of inputs [20, 21].

Model performance was assessed using the AUC of the receiver-operating characteristic (ROC) curves for the training and test sets. 95% confidence intervals were obtained for the AUC estimates using the variance of the AUC defined by Delong et al. [22] and the algorithm by Sun and Xu [23] and estimated with qnorm. Accuracy, sensitivity, specificity, negative predictive value (NPV), and positive predictive value (PPV) were assessed using the pROC package [24]. For calculating the sensitivity and specificity, the cutoff was set to 0.5, with predictions ≥ 0.5 as the good responder group. Feature importance was determined based on standardized LASSO coefficients (with larger magnitudes being of importance) and the mean decrease in Gini score (with higher decrease in Gini implying greater importance in partitioning the data into defined classes) for random forests. The importance of the most important feature was defined as 100, and the importance of the other features was reported relative to that feature. Calibration was assessed for the validation data using calibration plots and the Hosmer–Lemeshow test. Recalibration was performed by re-estimating the intercept and slope for the model prediction in the validation cohort [25].

A matrix model for “risk of good response to MTX” was created with a multivariate logistic regression model. The model was applied to the imputed data from all four RCTs and included baseline DAS28-ESR, ACPA status, and HAQ.

Additionally, the outcome of DAS28-ESR ≤ 3.2 at 24 weeks in patients who had DAS28-ESR > 3.2 at 12 weeks was investigated. We included all patients who had DAS28-ESR > 3.2 at 12 weeks to train a LASSO model. The model included the baseline variables used in the model with DAS28-ESR with the change in DAS28-ESR from baseline to 12 weeks as an additional predictor. A cutpoint analysis was performed to determine the cutpoint values for the following: (1) change in DAS28-ESR from baseline to week 12 for investigating the outcome of DAS28-ESR < 3.2 at 24 weeks among patients who had DAS28-ESR > 3.2 at 12 weeks and (2) baseline DAS28-ESR and HAQ for investigating the outcome of good response to MTX at 24 weeks among patients from the four RCTs [26]. *p*-values < 0.05 were considered statistically significant. Analyses were performed in RStudio version 1.2.5033 with R version 3.5.2. This study was considered exempt by the institutional review board of Mayo Clinic (IRB #17–002,593).

## Results

Seven-hundred seventy-five patients with available DAS28-ESR at baseline and 12 and 24 weeks were included (Table 1). All patients were MTX-naïve at trial enrollment, and RA duration was ≤ 24 months in 91% of patients.

**Table 1:** Baseline comparisons between responder groups and training/test sets. Baseline demographic and clinical characteristics were compared (a) between the good and poor responders defined using latent class mixed modeling and (b) between the unimputed training and test sets

	**Responder group comparisons**			**Training/test set comparisons**			**Total** **(*n* = 775)**
	**Good responders (*n* = 510)**	**Poor responders (*n* = 265)**	*p*-value*	**Training set (*n* = 365)**	**Test set (*n* = 410)**	*p*-value*	
**Age**, years	50 (40, 58) (*n* = 371)	50 (41, 57) (*n* = 237)	0.66	50 (40, 57)	50 (40, 59) (*n* = 243)	0.40	50 (40, 58) (*n* = 608)
**Female**, *n* (%)	402 (78.8)	215 (81.1)	0.45	286 (78.4)	331 (80.7)	0.41	617 (79.6)
**Race:** White, *n *(%)	269 (52.7)	157 (59.2)	0.08	239 (65.5)	187 (45.6)	< 0.001	426 (55)
**DAS28-ESR**	6.5 (5.9, 7.2)	7.6 (6.9, 8.1)	< 0.001	7.1 (6.4, 7.8)	6.7 (6.0, 7.4)	< 0.001	6.9 (6.2, 7.6)
**TJC28**	14 (9, 20)	21 (16, 26)	< 0.001	18 (12, 24)	15 (10, 22)	< 0.001	16 (11, 23)
**SJC28**	10.5 (7, 15)	15 (11, 22)	< 0.001	13 (9, 19)	11 (8, 16)	< 0.001	12 (8, 17)
**ESR**, mm/h	42.5 (31, 64)	63 (42, 88)	< 0.001	57 (38, 81)	42.5 (32, 64)	< 0.001	49 (34, 74.5)
**PtGA**	65 (50, 79)	76 (61, 88)	< 0.001	71 (55, 83)	67 (50, 82)	< 0.001	69 (52, 83)
**CRP**, mg/L	2.3 (0.9, 5.0) (*n* = 509)	3.1 (1.4, 7.2) (*n* = 263)	< 0.001	2.4 (1.3, 4.4) (*n* = 362)	2.7 (0.8, 8.9)	0.25	2.5 (1.0, 6.2) (*n* = 772)
**PhGA**	66 (52, 76) (*n* = 509)	72 (62, 83)	< 0.001	70 (56, 80) (*n* = 364)	68 (53.2, 77)	0.034	68.5 (55, 78) (*n* = 774)
**RF positive**, *n *(%)	463 (91.1) (*n* = 508)	241 (90.9)	0.93	329 (90.1)	375 (91.9) (*n* = 408)	0.39	704 (91.1) (*n* = 773)
**ACPA positive**, *n *(%)	447 (88.3) (*n* = 506)	230 (87.8) (*n* = 262)	0.82	325 (90.3) (*n* = 360)	352 (86.3) (*n* = 408)	0.09	677 (88.2) (*n* = 768)
**Glucocorticoids use**, *n *(%)	124 (46.4) (*n* = 267)	56 (36.4) (*n* = 154)	0.044	87 (48.9) (*n* = 178)	93 (38.3) (*n* = 243)	0.030	180 (42.8) (*n* = 421)
**HAQ**	1.6 (1.1, 2.0) (*n* = 463)	2.0 (1.6, 2.4) (*n* = 238)	< 0.001	2.0 (1.6, 2.2) (*n* = 294)	1.6 (1.1, 2.0) (*n* = 407)	< 0.001	1.8 (1.2, 2.1) (*n* = 701)
**RA duration**, months	3.7 (1.5, 7.9)	5.3 (2.2, 13.4)	< 0.001	6.9 (3.6, 17.2)	2.2 (1.0, 5.7)	< 0.001	4.1 (1.7, 9.2)
**Good Responders**, *n *(%)				199 (54.5)	311 (75.9)	< 0.001	

Median (interquartile range) are reported unless noted otherwise

*Abbreviations*: *ACPA*, anti-citrullinated protein antibody, *CRP*, C-reactive protein, *DAS28-ESR*, disease activity score including 28-joint counts and ESR, erythrocyte-sedimentation rate, *HAQ*, Health Assessment Questionnaire, *PtGA*, Patient’s Global Assessment of Disease Activity, *PhGA*, Physician’s Global Assessment of Disease Activity, *RA*, rheumatoid arthritis, *RF*, rheumatoid factor, *SJC28*, swollen joint count, *TJC28*, 28-tender joint count

^*^p-values are reported from the Kruskal–Wallis rank sum test for continuous data and Pearson’s chi-squared test for categorical data

### Latent class modeling of MTX response

**Fig. 1:**
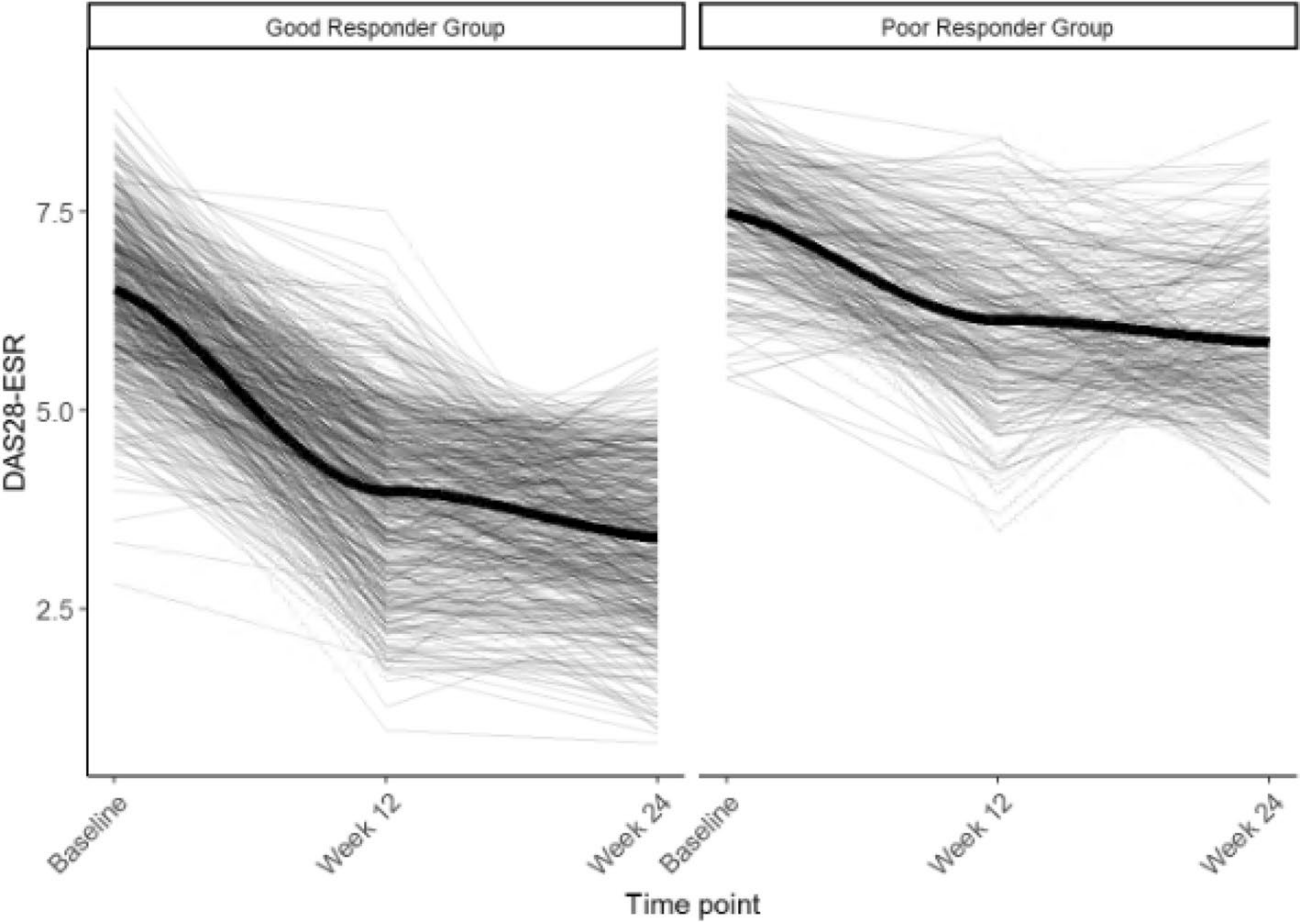
Two patient class trajectories identified with latent class modeling of DAS28-ESR (*N* = 775)

A comparison of five different latent class mixed models (Additional file 1: Table S2) showed the model with two patient classes with similar trajectories to be most promising with the lowest Akaike Information Criterion (AIC) and sample size adjusted Bayesian information criterion (SABIC) values (Fig. 1). We identified two distinct patient classes: The class 1 “good responder” group had a mean 3.1 (SD 1.3) improvement in DAS28-ESR from baseline to 24 weeks (*n* = 510), and the class 2 “poor responder” group had a mean 1.6 (SD 1.2) improvement in DAS28-ESR from baseline to 24 weeks (*n* = 265).

### Baseline sociodemographics and clinical factors and their association with DAS28-ESR-based clusters

No significant differences were observed between the good and poor responders in age, sex, race, RF status, and ACPA status (Table 1). The poor responders had significantly higher DAS28-ESR at baseline, SJC28, TJC28, ESR, CRP, PtGA, PhGA, and HAQ and longer RA duration, but lower percentage of glucocorticoid users at baseline, compared to the good responders (Table 1).

### Training and test data

The training set contained 47% (*n* = 365) of the total dataset from two randomly selected RCTs, and the test set consisted of 53% (*n* = 410) of the total dataset from the remaining two RCTs. Comparing the training and test sets, there were significant differences in race, DAS28-ESR at baseline, SJC28, TJC28, ESR, PtGA, PhGA, glucocorticoid use, HAQ, RA duration, and proportion of good responders; no significant differences were observed in age, sex, CRP, RF status, and ACPA status (Table 1). Glucocorticoid use (45.7%) and age (21.5%) had the highest percentages of missing data; the remaining characteristics had percentages of missing data ranging from 0 to 9.5%.

### Model performances on training and test sets

**Fig. 2:**
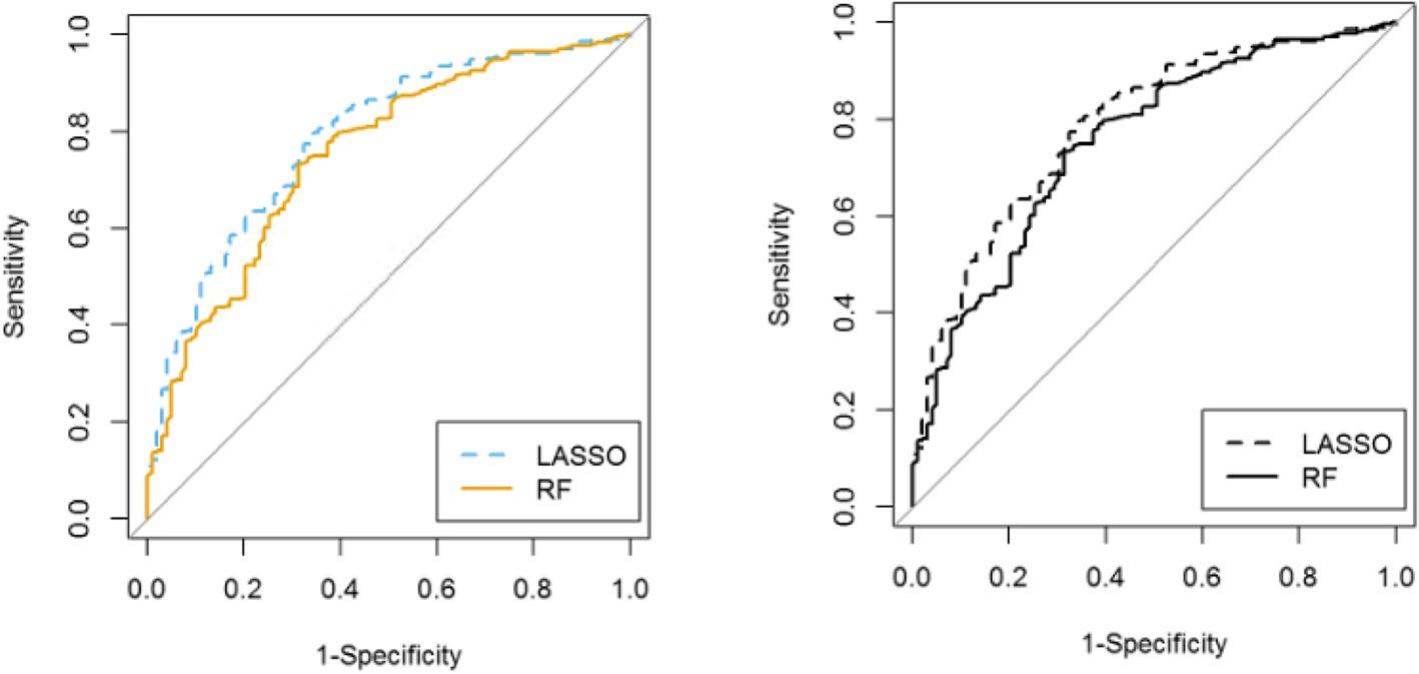
Receiver-operating characteristic (ROC) curves of algorithms validated on the test set for the “DAS28-ESR” model (*N* = 410). The area under the curve (AUC) for the least absolute shrinkage and selection operator (LASSO; dashed) and random forests (RF; solid) methods validated on the test set were 0.79 and 0.68, respectively. LASSO, least absolute shrinkage and selection operator; RF, random forests

Performance of algorithms on the DAS28-ESR model and the model with components of DAS28-ESR at baseline were comparable (Table 2). The LASSO algorithm performed better than the random forests, providing an AUC of 0.79 (95% CI 0.74, 0.84) externally validated on the test set for both models. High sensitivity values observed in both models using the test set (sensitivity = 0.83 for the DAS28-ESR model; sensitivity = 0.86 for the components of DAS28-ESR model) implied both models performed well in identifying those who were good responders to MTX. However, calibration was poor in the validation set. Following recalibration, the calibration was good with confidence intervals overlapping the identity line for all 10 deciles, but the Hosmer–Lemeshow test remained significant indicating poor calibration (*p* < 0.001). The ROC curves for the LASSO and random forests validated on the test set for the DAS28-ESR model are illustrated in Fig. 2. Confusion matrices for the training and test data sets for the DAS28-ESR model for LASSO and random forests are provided in Additional file 1: Table S3.

**Table 2:** Model performances on the training (*N* = 365) and test (*N* = 410) sets. Two models were investigated. The “DAS28-ESR” model consisted of baseline DAS28-ESR, age, sex, race, RA duration, RF status, ACPA status, glucocorticoid use, and HAQ score. The “components of DAS28-ESR” model consisted of TJC28, SJC28, ESR, PtGA, CRP, PhGA, age, sex, race, RA duration, RF status, ACPA status, glucocorticoids use, and HAQ score. High sensitivity values observed in both LASSO models using the test set implied both models performed well in identifying those who were good responders to methotrexate. For calculating sensitivity and specificity, the cutoff was set to 0.5, with predictions greater than or equal to 0.5 classified as the “good” responder group

Algorithm	Model	Training set (*N* = 365, 2 RCTs)						Test set (*N* = 410, 2 RCTs)					
		**AUC** **(95% CI)**	**Sensitivity**	**Specificity**	**Accuracy**	**PPV**	**NPV**	**AUC** **(95% CI)**	**Sensitivity**	**Specificity**	**Accuracy**	**PPV**	**NPV**
LASSO	DAS28-ESR	0.76 (0.71, 0.81)	0.73	0.66	0.70	0.72	0.67	0.79 (0.74, 0.84)	0.83	0.61	0.78	0.87	0.53
	Components of DAS28-ESR	0.77 (0.72, 0.81)	0.75	0.65	0.70	0.72	0.68	0.79 (0.74, 0.84)	0.86	0.58	0.79	0.86	0.56
Random forests	DAS28-ESR	0.96 (0.94, 0.98)	0.97	0.96	0.96	0.97	0.96	0.68 (0.62, 0.73)	0.81	0.55	0.75	0.85	0.48
	Components of DAS28-ESR	1	1	1	1	1	1	0.68 (0.63, 0.74)	0.77	0.60	0.73	0.86	0.45

*Abbreviations*: *AUC*, area under the curve; *DAS28-ESR*, Disease Activity Score including 28-joint counts and erythrocyte sedimentation rate, *LASSO*, least absolute shrinkage and selection operator, *NPV*, negative predictive value, *PPV*, positive predictive value, *RCTs*, randomized clinical trials

### Characteristics importance

**Fig. 3:**
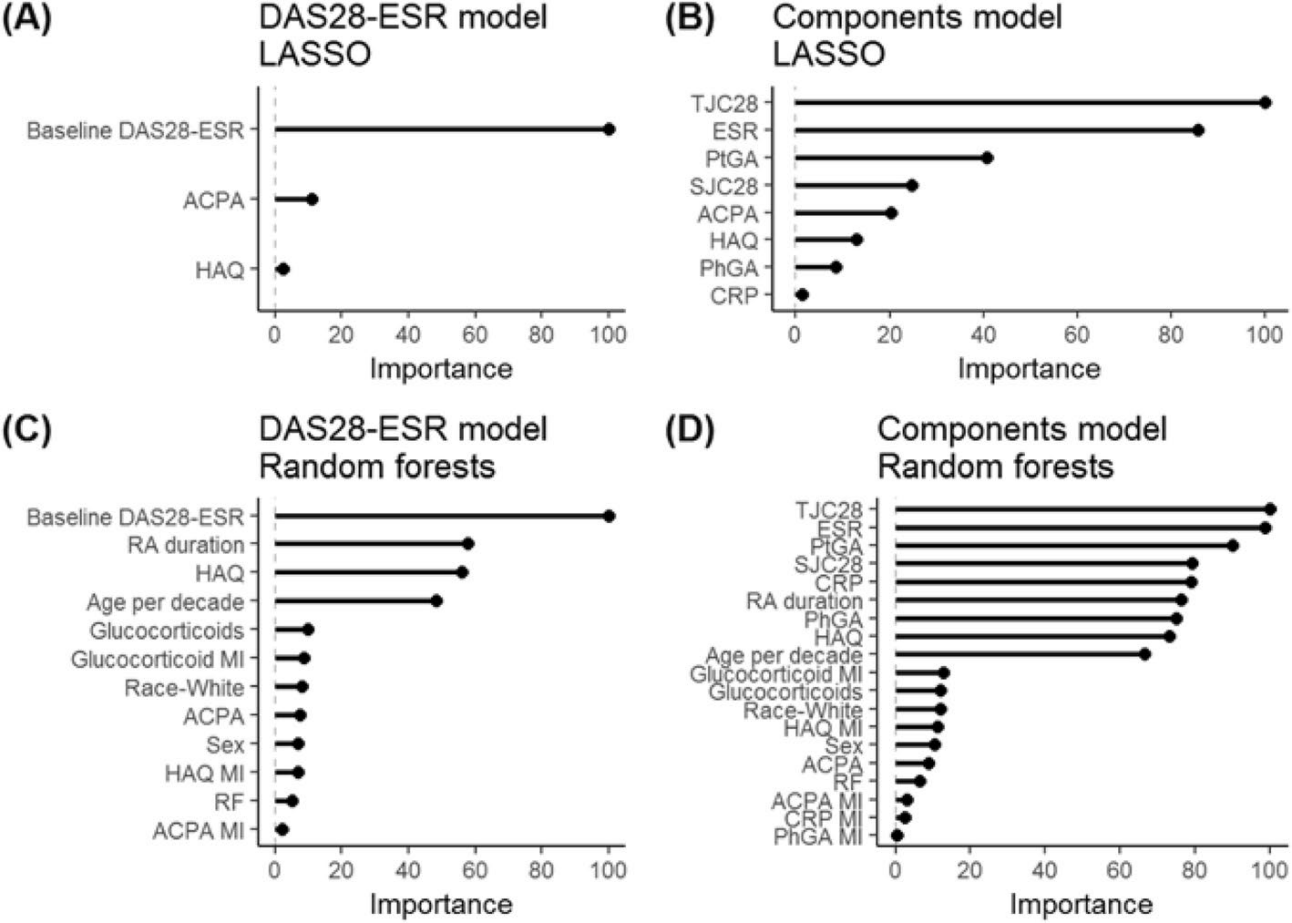
Feature importance plots of characteristics for **A** LASSO and **B** random forests. Feature importance plots for **A** the DAS28-ESR model with LASSO algorithm, **B** the components of DAS28-ESR model with LASSO algorithm, **C** the DAS28-ESR model with random forests methods, and **D** the components of DAS28-ESR model with random forests methods are provided below. Feature importance was determined based on standardized LASSO coefficients and the Gini score for random forests. The most important feature was set to 100, and the rest is relative to that feature. DAS28ESR, Disease Activity Score with 28-joint count with erythrocyte sedimentation rate; RA, rheumatoid arthritis; SJC66, 66 Swollen Joint Count; ESR, erythrocyte sedimentation rate; ACPA, anti-citrullinated protein antibodies; TJC68, 68 Tender Joint Count; CRP, C-reactive protein; HAQ, Health Assessment Questionnaire Score; PhGA, Physician’s Global Assessment of Disease Activity; PtGA, Patient’s Global Assessment of Disease Activity; MI, missing indicator

Feature importance plots of baseline features for LASSO and random forests are shown in Fig. 3. Baseline DAS28-ESR was shown to be among the top three predictors with high importance in both LASSO and random forests. Using LASSO methods, baseline DAS28-ESR, ACPA status, and HAQ were important predictors of good response to MTX. Concordantly, using random forests, the top four predictors of good response to MTX were baseline DAS28-ESR, RA duration, HAQ, and age. The remaining characteristics were not predictive of good response to treatment with MTX (Fig. 3).

### Multivariate risk profiling (matrix prediction model)

The cutpoint analysis for baseline DAS28-ESR and HAQ resulted in the optimal cutpoint values of 7.4 and 2, respectively. The predictive probability (summarized as percentages with 95% CIs) of achieving a good response to MTX at 24 weeks based on the combination of DAS28-ESR, ACPA status, and HAQ at baseline is shown in Table 3.

**Table 3:** Matrix prediction model. Probability of achieving a good response to methotrexate at 24 weeks

		**HQA**			
		≤ 2	> 2		
**DAS28-ESR**	≤ 7.4	80.1 (76.4, 83.8)	77.3 (70.6, 84)	Positive	**ACPA status**
		77.1 (68.6, 85.6)	74.1 (63.3, 84.9)	Negative	
	> 7.4	40.3 (32.1, 48.5)	36.5 (29.3, 43.6)	Positive	
		36.2 (23.3, 49.1)	32.5 (20.9, 44.1)	Negative	

The number in each cell represents the percentage and 95% CI of achieving the outcome, based on the combination of predictors at baseline. *DAS28-ESR*, Disease Activity Score with 28-joint count with erythrocyte sedimentation rate, *HAQ*, Health Assessment Questionnaire; *ACPA*, anti-citrullinated protein antibody

### Subset analysis

Six-hundred fifty-one patients had DAS28-ESR > 3.2 at 12 weeks and were included in the subset analysis investigating the outcome of DAS28-ESR ≤ 3.2 at 24 weeks. Summary and comparison of baseline characteristics between patients with DAS28-ESR ≤ 3.2 at 24 weeks (*n* = 122) and patients with DAS28-ESR > 3.2 at 24 weeks (*n* = 529) are provided in Additional file 1: Table S4. There were significant differences between the two groups in age, change in DAS28-ESR at week 12 from baseline, baseline DAS28-ESR, TJC28, ESR, PtGA, HAQ, and RA duration. No significant differences were observed between the two groups in sex, race, SJC28, CRP, PhGA, RF status, ACPA status, and glucocorticoid use (Additional file 1: Table S4). The LASSO algorithm performed well with AUC 0.80 (95% CI 0.76, 0.84). With 0.24 as the cutoff to maximize sensitivity and specificity, sensitivity was 0.70, and specificity was 0.78. The top two characteristics with their odds ratio (OR) furthest from one were change in DAS28-ESR at week 12 from baseline (OR = 0.35) and baseline DAS28-ESR (OR = 0.43). Improvement in DAS28-ESR by at least 1 point in the first 12 weeks was associated with achieving DAS28-ESR ≤ 3.2 at 24 weeks.

## Discussion

Approaches for dealing with vast heterogeneity in response to MTX among individual patients with RA are insufficiently addressed in the current treatment guidelines, and systematic patient-tailored tools to personalize early RA management are lacking [27, 28]. This is one of the first studies using ML methods to identify the latent trajectories of DAS28-ESR over 24 weeks in new users of MTX with high RA disease activity at baseline. The clinical phenotype which we defined as “good responders” comprising lower baseline DAS28-ESR score and its individual components, positive ACPA, and lower baseline HAQ score ranked in the order of predictive importance. “Good responders” at 24 weeks accounted for 66% of all patients, consistent with previously reported rate of response to MTX at this time point [29]. The finding that lower baseline disease activity and better functional status at baseline are predictive of “good responders” to MTX is not unexpected and is concordant with previous studies [11, 29, 30]. In addition, we have provided cut points and a matrix prediction model for a good response to MTX: patients with DAS28-ESR ≤ 7.4, positive ACPA, and HAQ ≤ 2 at baseline have an 80% probability of being good responders, while having DAS28-ESR and HAQ above these cutoffs in combination with ACPA negative status results in only 33% probability of good response. Consistent with the prediction model, DAS28-ESR was the most important predictor in multivariate risk profiling. These findings can inform clinical decision-making and patient-physician discussions about the likelihood of treatment success in patients initiating MTX. By quantifying the probability of response to MTX using baseline clinical data, our findings may facilitate consideration of the use of biologics or targeted synthetic DMARDs in treatment-naïve patients with RA and poor prognostic factors, a scenario of RA treatment which is not specifically addressed in the current ACR or EULAR guidelines [27, 28]. Further studies employing our model will be needed to assess the clinical utility of our model and its implications for clinical practice.

The components of our model were very similar to the Rheumatoid Arthritis Medication Study (RAMS) [29], except in that study lower DAS28 was paradoxically associated with non-response to MTX. This was likely due to the definition of non-response in the RAMS study requiring at least 0.6 units decline in DAS28 score which is more likely to happen in patients with higher DAS28 at baseline. In our study, the classes of patients were defined algorithmically based on DAS28 trajectory, and the prediction was for the class of responders rather than for the change in DAS28, consistent with the association between lower DAS28 and achieving a “state” outcome (i.e., remission or low disease activity) in prior studies [11].

Unlike the previous studies which evaluated the outcomes at 12 months [11, 26], we focused on the earlier time points (i.e., 3 and 6 months) as the most critical “window of opportunity” for decision-making regarding the future management plan in early RA, consistent with the American College of Rheumatology recommendations [31]. Indeed, RA disease duration was one of the top predictors in our random forests, consistent with the established knowledge that delay in treatment is an adverse prognostic factor in achieving treatment targets in RA [4, 5, 9].

In line with our finding of ACPA positivity as a predictor of the “good responders” class, higher likelihood of early response (i.e., 4 months) to treatment with MTX in seropositive patients with abundant autoantibodies including ACPA was reported in the induction therapy with MTX and prednisone in RA or very early arthritic disease (IMPROVED) study [32]. However, no such association was found for response at 1 year in the IMPROVED study. In the RAMS, not being RF-positive was associated with non-response to MTX at 6 months [29]. It has been suggested that the presence of multiple autoantibodies at baseline may reflect a more active autoimmune response which is more susceptible to suppression by MTX in the initial stages, but not in later stages [32]. Indeed, ACPA positivity has been associated with lower rates of drug-free remission in RA in several large studies [33–36], potentially indicating the persistence of a population of ACPA IgG-producing autoreactive B-cells that is resistant to therapy and accounts for the inability to achieve drug-free remission in RA in the long run [32]. Given that ACPA and RF seropositivity can be helpful in informing response to treatment with other antirheumatic medications in RA (e.g., abatacept and rituximab), confirming the value of differential prediction of early response to MTX treatment in RA by serostatus in future studies may help to further refine the approach to the management of early RA [37, 38].

Among the individual components of DAS28, TJC28, ESR, PtGA, and SJC28 were among the top five predictors of “good responders.” Concordantly, higher TJC28 was an independent predictor of non-response to MTX at 6 months in the RAMS [29]. DAS28, TJC28, HAQ, and ESR were among the top predictors of insufficient response to DMARD treatment using LASSO and random forests in a recent study from The Netherlands [16]. While at least one-third of patients in the study by Gosselt et al. used MTX in combination with sulfasalazine or hydroxychloroquine, our study included patients on MTX who were not on other DMARDs.

All four RCTs included in the study used an up-titration scheme for MTX, maximizing the dose to 20–25 mg/week, thus decreasing the possibility of non-response due to underdosing. Good responders were more likely to use glucocorticoids at baseline, concordant with the data that MTX monotherapy with glucocorticoid bridging can be clinically beneficial in achieving treatment response in RA [39].

Sociodemographic characteristics were among the predictors in random forests models but were not retained in the LASSO models. Sociodemographic and economic parameters have been associated with the persistence of MTX treatment in prior studies using ML methods, but these models are not directly comparable to the prediction of response to MTX (i.e.., MTX efficacy) in our study [15]. There is some ambiguity in the predictive role of sociodemographics for MTX response, mainly with regard to the predictive role of the female sex which was found to be associated with lower likelihood of response in some but not other studies [5, 9, 29, 40, 41]. This discrepancy may be at least in part due to the use of disease activity metrics including ESR without accounting for age- and sex-specific cutoffs for ESR which may bias the assessment of treatment response. In this study, we were not able to retrieve CRP measures for 12 and 24 months but used DAS28-ESR which was an outcome measure used in the included RCTs. Using disease activity metrics that do not include ESR in future studies may help further refine our understanding of the predictive value of sex in response to antirheumatic treatments.

In this study, we successfully performed external validation of our models which has not been done in the previous studies [16, 29]. Importantly, models with individual components of DAS28-ESR had similar performance to models with DAS28-ESR score, supporting the construct validity of the DAS28-ESR measure. The modest discrimination value (AUC 0.79) of our LASSO models is non-inferior to the previous models including clinical predictors of response to MTX in RA [16, 29] and dictates the need for additional biomarkers aiming at the improved performance of individualized predictive models. Studies are underway by our group to augment clinical predictors with genomic, metabolomic, and microbiome data [42, 43].

Among patients who had DAS28-ESR > 3.2 at 12 weeks, the majority (81%) did not achieve DAS28-ESR ≤ 3.2 at week 24 which is concordant with studies showing that in over 75% of patients the trajectory of response or non-response to MTX is consistent between 3 and 6 months [44]. Extending this prior knowledge, we have identified and ranked in the order of importance predictors of achieving low disease activity by DAS28-ESR at 24 weeks among those who did not achieve DAS28-ESR at 12 weeks. A steeper decline in DAS28-ESR (i.e., at least 1 point improvement in DAS28-ESR) from baseline to week 12 has been identified as the top predictor of response in this group, which can be used in clinical decision-making and discussions regarding the likelihood of achieving low disease activity at 24 weeks among patients who have not achieved low disease activity at 12 weeks. This finding applies to patients who continue MTX monotherapy for the first 24 weeks of treatment. Further studies are needed to understand the relevance of this initial improvement in DAS28-ESR for the prediction of response to therapy escalation at week 12. Other predictors of DAS28-ESR ≤ 3.2 at week 24 in this group included lower baseline DAS28-ESR, younger age, positive ACPA, shorter RA duration, and lower baseline HAQ, in line with our main prediction model.

There are several limitations to our study. First, we had no information about some risk factors that have been previously linked to lower likelihood of treatment response, i.e., low socioeconomic status, smoking, obesity, mental and physical comorbidities, and non-adherence to treatment [29, 45, 46]. The addition of these risk factors would be expected to further refine the prediction. Second, data for some variables (primarily ACPA and glucocorticoid use) were missing for a proportion of patients. Imputation of the missing values in predictor data and addition of missing data indicator variables in the statistical models can help minimize this shortcoming. The missing data indicator did not appear to be among the significant predictors in the main LASSO models with DAS28 and its components but was present in the random forests models, requiring caution in interpreting the results. Third, most patients included in this study had high RA disease activity, reflective of the RCT study population. Thus, the results may not be generalizable to patients with low-moderate RA disease activity at baseline. Future studies identifying predictors of response to MTX in patients with low-moderate RA disease activity should help to further inform early RA management.

The main strengths of the study include the use of high-quality longitudinal data of treatment-naïve patients with active RA from 4 RCTs, forming a large training dataset with an independent testing set for external validation. We used a data-driven ML approach combining sociodemographic and clinical data to identify distinct patient trajectories based on DAS28-ESR. The model included clinician-friendly, easily available clinical measures and was externally validated in an independent test set.

In conclusion, we have developed and externally validated a prediction model for response to MTX monotherapy within 24 weeks in DMARD-naïve patients with high RA disease activity at baseline, providing variably weighted predictive clinical features and defined cutoffs for clinical decision-making. The trajectory of DAS28-ESR change over 24 weeks in patients with high RA disease activity at baseline who are starting MTX can be predicted by baseline DAS28-ESR, ACPA status, and HAQ score. These parameters should be considered as part of the clinical decision-making process when initiating MTX in DMARD-naïve patients with RA. For example, a patient with RA who is ACPA-positive and has HAQ ≤ 2 and DAS28-ESR ≤ 7.4 would have a good predictive probability of achieving a good response to MTX therapy. In contrast, a patient with DAS28-ESR > 7.4, who is ACPA-negative and has HAQ > 2, would be predicted to have a poor response to MTX treatment, and a more aggressive treatment regimen (e.g., addition of a biologic agent) can be discussed. Patients with over 1 unit decline in DAS28-ESR within the first 12 weeks of treatment who have not achieved low disease activity by week 12 may be more likely to achieve low disease activity at 24 weeks. This information may allow physicians to tailor treatment approaches based on the likelihood of treatment response and can help improve RA disease outcomes and patient satisfaction by better managing patients’ expectations.

## Supplementary Information

**Additional file 1:** **Table S1.** The randomized clinical trials and patients included in the study. **Table S2.** Model comparison. **Table S3.** Confusion matrices for the training and test data sets. **Table S4.** Baseline comparisons between 24-Week responders and non-responders.

## Data Availability

The data used and analyzed in the current study are available through the Clinical Study Data Request Consortium (CSDR) via the Vivli Center for Global Clinical Research Data. Restrictions apply to the availability of these data, which were used under agreement for the current study, and therefore are not publicly available. However, data are available from the authors upon reasonable request and with permission from Vivli Center.

## Abbreviations

ACPA: Anti-citrullinated protein antibodies
AIC: Akaike Information Criterion
AUC: Area under the curve
CRP: C-reactive protein
CSDR: Clinical Study Data Request Consortium
DAS28-ESR: Disease Activity Score including 28 joints, erythrocyte sedimentation rate
DMARD: Disease-modifying antirheumatic drug
ESR: Erythrocyte sedimentation rate
HAQ: Health Assessment Questionnaire
IMPROVED: Induction therapy with MTX and prednisone in RA or very early arthritic disease study
LASSO: Least absolute shrinkage and selection operator
ML: Machine learning
MTX: Methotrexate
RA: Rheumatoid arthritis
RCT: Randomized clinical trial
PtGA: Patient global assessment of disease activity
PhGA: Physician global assessment of disease activity
RAMS: Rheumatoid Arthritis Medication Study
RF: Rheumatoid factor
SABIC: Sample size-adjusted Bayesian information criterion
SJC 28: Swollen joint count of 28 joints
TJC28: Tender joint count of 28 joints
