# Supplementary material for: Clinical predictors of response to methotrexate in patients with rheumatoid arthritis: a machine learning approach using clinical trial data
Source: Arthritis Res Ther. 2022 Jul 1;24:162. doi: 10.1186/s13075-022-02851-5 (PMC9248180; doi:10.1186/s13075-022-02851-5)
Supplement: Supplementary file 1 — Additional file 1: Table S1. The randomized clinical trials and patients included in the study. Table S2. Model comparison. Table S3. Confusion matrices for the training and test data sets. Table S4. Baseline comparisons between 24-Week responders and non-responders. [file 13075_2022_2851_MOESM1_ESM.docx]

# Supplementary Table A. The randomized clinical trials and patients included in the study

Studies involving MTX-naïve patients with RA who were randomized to placebo plus MTX were considered in this study. Patients with available Disease Activity Score with 28-joint count (DAS28) with erythrocyte sedimentation rate (ESR) at baseline, 12, and 24 weeks were included.

Abbreviations: RCT: Randomized clinical trial; MTX: methotrexate; RA: Rheumatoid arthritis; DAS28: Disease Activity Score with 28-joint count; PO: per os (by mouth)

| **RCT** | **MTX dose, mg/week** | **RA duration at inclusion** | **Country** | **Year(s) of RCT** | **Placebo plus MTX** | **DAS28 at baseline, 12 and 24 weeks available** | **Analysis cohort** |
| --- | --- | --- | --- | --- | --- | --- | --- |
| NCT00299104 | 7.5 mg PO escalating by 2.5 mg a week every 1-2 weeks to achieve: 15 mg per week by Week 4 and 20 mg per week by Week 8 | ≥ 2 months | USA, Europe | 2006-2008 | 249 | 178 | 178 |
| NCT00485589 | 7.5 mg PO, increasing to 20 mg by week 8 | 3 months-5 years | USA, Europe | 2007-2010 | 207 | 187 | 187 |
| NCT01007435 | 7.5 mg PO. If a patient had swollen or tender joints, the dose was increased to 15 mg and 20 mg weekly, at the Week 4 and Week 8 visits | ≤ 2 years | USA, Europe, Canada, Thailand | 2009-2012 | 285 | 243 | 243 |
| NCT01519791 | 10 mg PO, escalated by 5 mg every 2 Weeks such that the maximum dosage of 25 mg per Week is achieved by Week 6 to Week 8 | less than 1 year | USA, Europe | 2012-2014 | 217 | 167 | 167 |
| Total | | | | | 958 | 775 | 775 |

#

# Supplementary Table B. Model comparison

Model comparison of five different latent class models

Abbreviations: G=number of classes; loglik=Log-likelihood; npm=number of parameters; AIC=Akaike information criterion; BIC=Bayesian information criterion; SABIC=Sample size adjusted BIC

|  | **G** | **loglik** | **conv** | **npm** | **AIC** | **BIC** | **SABIC** | **entropy** | **%class1** | **%class2** | **%class3** | **%class4** | **%class5** |
| --- | --- | --- | --- | --- | --- | --- | --- | --- | --- | --- | --- | --- | --- |
| m1 | 1 | -3792.6 | 1 | 6 | 7597.2 | 7625.1 | 7606.1 | 1.0 | 100.0 | NA | NA | NA | NA |
| m2 | 2 | -3782.9 | 1 | 9 | 7583.8 | 7625.7 | 7597.1 | 0.51 | 65.8 | 34.2 | NA | NA | NA |
| m3 | 3 | -3781.2 | 1 | 12 | 7586.4 | 7642.3 | 7604.1 | 0.51 | 35.9 | 45.3 | 18.8 | NA | NA |
| m4 | 4 | -3780.1 | 2 | 15 | 7590.3 | 7660.1 | 7612.4 | 0.58 | 19.9 | 41.7 | 29.5 | 8.9 | NA |
| m5 | 5 | -3780.1 | 2 | 18 | 7596.2 | 7680.0 | 7622.8 | 0.53 | 15.9 | 0.6 | 45.2 | 29.3 | 9.0 |

# Supplementary Table C. Confusion matrices for the training and test data sets

Confusion matrices for the training and test data sets for LASSO and random forest models looking at the ‘DAS28-ESR’ model with baseline DAS28-ESR, age, sex, race, RA duration, RF status, ACPA status, glucocorticoids use, and HAQ score. (Inaccurate predictions are highlighted in gray.)

|  | | **Training set (N=365, 2 RCTs)** | | | | **Test set (N=410, 2 RCTs)** | | | |
| --- | --- | --- | --- | --- | --- | --- | --- | --- | --- |
|  | | **LASSO** | | **Random forest** | | **LASSO** | | **Random forest** | |
| **Actual values** | | **High** | **Low** | **High** | **Low** | **High** | **Low** | **High** | **Low** |
| **Predicted values** | **High** | 146 | 56 | 193 | 7 | 258 | 39 | 252 | 45 |
|  | **Low** | 53 | 110 | 6 | 159 | 53 | 60 | 59 | 54 |
| Abbreviations:  LASSO: Least absolute shrinkage and selection operator; RCTs: randomized clinical trials | | | | | | | | | |

#

# Supplementary Table D. Baseline comparisons between 24-Week responders and non-responders

Baseline demographic and clinical characteristics were compared between the patients who had DAS28-ESR > 3.2 at 12 weeks with and without DAS28-ESR ≤ 3.2 at 24 weeks.

|  | **DAS28-ESR ≤ 3.2**  **at 24 weeks**  **(n=122)** | **DAS28-ESR > 3.2**  **at 24 weeks**  **(n=529)** | **Total**  **(n=651)** | **p-value*** |
| --- | --- | --- | --- | --- |
| **Age***, years* | 44 (35.5, 55) (n=87) | 51 (41, 59) (n=438) | 50 (40, 58) (n=525) | 0.004 |
| **Female**, *n (%)* | 98 (80.3) | 420 (79.4) | 518 (79.6) | 0.82 |
| **Race: White**, *n (%)* | 61 (50) | 297 (56.1) | 358 (55) | 0.22 |
| **Change in DAS28-ESR at Week 12 from baseline** | -2.4 (-3.0, -1.7) | -1.7 (-2.5, -1.0) | -1.9 (-2.6, -1.0) | <0.001 |
| **DAS28-ESR** | 6.7 (6.0, 7.2) | 7.1 (6.4, 7.8) | 7.0 (6.3, 7.7) | <0.001 |
| **TJC28** | 15.5 (10.2, 21) | 18 (12.0, 24) | 17 (11, 23.5) | 0.015 |
| **SJC28** | 12 (8, 16.8) | 13 (9, 18) | 13 (8.5, 18) | 0.17 |
| **ESR***, mm/h* | 42 (30, 57.8) | 56 (37, 80) | 53 (35, 78) | <0.001 |
| **PtGA** | 63 (52, 75) | 72 (55, 84) | 70 (53.5, 83) | 0.002 |
| **CRP***, mg/L* | 2.4 (1, 6.7) | 2.8 (1.2, 6.6) (n=526) | 2.8 (1.1, 6.6) (n=648) | 0.48 |
| **PhGA** | 69 (50.2, 78) | 70 (57, 80) (n=528) | 70 (56, 79) (n=650) | 0.14 |
| **RF positive**, *n (%)* | 110 (90.2) | 482 (91.3) (n=528) | 592 (91.1) (n=650) | 0.69 |
| **ACPA positive**, *n (%)* | 108 (89.3) (n=121) | 462 (88.2) (n=524) | 570 (88.4) (n=645) | 0.74 |
| **Glucocorticoids use**, *n (%)* | 33 (46.5) (n=71) | 118 (41) (n=288) | 151 (42.1) (n=359) | 0.40 |
| **HAQ** | 1.8 (1.2, 2) (n=107) | 1.9 (1.5, 2.2) (n=481) | 1.9 (1.4, 2.2) (n=588) | 0.007 |
| **RA duration***, months* | 3.7 (1.7, 7.4) | 4.5 (1.9, 10.9) | 4.4 (1.8, 10.1) | 0.036 |
| Median (interquartile range) are reported unless noted otherwise.  Abbreviations: DAS28-ESR: Disease activity score including 28-joint counts and erythrocyte-sedimentation rate; TJC28: 28-Tender joint count; SJC28: Swollen joint count; ESR: Erythrocyte-sedimentation rate; PtGA: Patient’s Global Assessment of Disease Activity; CRP: C-reactive protein; PhGA: Physician’s Global Assessment of Disease Activity; RF: Rheumatoid Factor; ACPA: Anti-citrullinated protein antibody; HAQ: Health assessment questionnaire; RA: Rheumatoid arthritis.  *P-values are reported from the Kruskal-Wallis rank sum test for continuous data and Pearson’s Chi-squared test for categorical data. | | | | |
